# Global Gene Expression Analysis Reveals Crosstalk between Response Mechanisms to Cold and Drought Stresses in Cassava Seedlings

**DOI:** 10.3389/fpls.2017.01259

**Published:** 2017-07-18

**Authors:** Shuxia Li, Xiang Yu, Zhihao Cheng, Xiaoling Yu, Mengbin Ruan, Wenbin Li, Ming Peng

**Affiliations:** ^1^Institute of Tropical Bioscience and Biotechnology, Chinese Academy of Tropical Agricultural Sciences Haikou, China; ^2^National Key Laboratory of Plant Molecular Genetics and National Center of Plant Gene Research, Institute of Plant Physiology and Ecology, Shanghai Institutes for Biological Sciences, Chinese Academy of Sciences Shanghai, China; ^3^Haikou Experimental Station, Chinese Academy of Tropical Agricultural Sciences Haikou, China

**Keywords:** cassava, cold stress, drought stress, RNA sequencing and transcriptome analysis, transcription factors, protein kinases, crosstalk

## Abstract

Abiotic stress negatively impacts cassava (*Manihot esculenta*) growth and yield. Several molecular mechanisms of plant response to cold and drought have been identified and described in the literature, however, little is known about the crosstalk of the responses of cassava to these two stresses. To elucidate this question, transcriptome analysis of cassava seedlings under cold or PEG-simulated drought stress treatment was performed. Our results showed that 6103 and 7462 transcripts were significantly regulated by cold and drought stress, respectively. Gene Ontology annotation revealed that the abscisic and jasmonic acid signaling pathways shared between the two stresses responses. We further identified 2434 common differentially expressed genes (DEGs), including 1130 up-regulated and 841 down-regulated DEGs by the two stresses. These co-induced or co-suppressed genes are grouped as stress signal perception and transduction, transcription factors (TFs), metabolism as well as transport facilitation according to the function annotation. Furthermore, a large proportion of well characterized protein kinases, TF families and ubiquitin proteasome system related genes, such as *RLKs*, *MAPKs*, *AP2/ERFBPs*, *WRKYs*, *MYBs*, E2 enzymes and E3 ligases, including three complexes of interacting proteins were shown as key points of crosstalk between cold and drought stress signaling transduction pathways in a hierarchical manner. Our research provides valuable information and new insights for genetically improving the tolerance of crops to multiple abiotic stresses.

## Introduction

Plant growth and productivity are severely affected by various abiotic stress factors such as cold and drought (Mahajan and Tuteja, 2005). These two stress factors prevent plants from reaching their full genetic potential and limit the crop yields around the world. In general, low temperature mainly results in plant surface lesion, discoloration, tissue break down, accelerated senescence, ethylene production and faster decay due to leakage of plant metabolites (Sharma et al., 2005; Solanke and Sharma, 2008). Cold stress also causes dehydration, mainly due to reduction in water uptake by roots and an impediment to close stomata (Solanke and Sharma, 2008). At freezing temperature, accumulation of ice in the intracellular spaces causes severe cellular dehydration and physical disruption of tissues (Solanke and Sharma, 2008). While drought stress has a profound adverse effect on leaf gas exchange properties, nutrient uptake, cell membrane stability, and proline and ascorbate contents (Lawlor and Cornic, 2002; Quan et al., 2015). Drought stress also exerts its detrimental effects by disrupting the ionic and osmotic equilibrium of the cell (Zhu, 2002). Plants are more vulnerable during germination and during early seedling growth to cold and drought stresses. Therefore, understanding the molecular mechanism of cold and drought stress signal transduction pathway is important for improving stress tolerance of crops.

Plant adaptation to cold and/or drought stress conditions is dependent upon the activation of cascades of molecular networks involved in signal transduction and the expression of specific stress-related genes and metabolites, with interactions and molecular crosstalk happening at several key control points (Chinnusamy et al., 2004). Therefore, understanding the common and specific signaling pathways of plants responding to cold and drought will shed new lights on the mechanisms of plants against various environmental stresses. Recently, some genes of Ca^2+^-signaling, abscisic acid (ABA)-signaling and nucleic acid pathways have been reported to be up-regulated in response to both cold and drought stresses indicating the presence of crosstalk between these pathways (An et al., 2012; Turyagyenda et al., 2013; Fu et al., 2016). Cytosolic Ca^2+^ is suggested as an important second messenger and which can be induced by both cold and drought stresses. Increase in cytosolic Ca^2+^ is sensed by calcium binding proteins (CaM), CaM domain-containing protein kinases (CDPKs), CBL-interacting protein kinases (CIPKs) and mitogen activated protein kinase (MAPKs), which transduce the signals to switch on transcriptional cascades (Solanke and Sharma, 2008; Huang et al., 2012). The overexpression of *NPK1* enhances freezing and drought tolerance in transgenic maize (Shou et al., 2004; Solanke and Sharma, 2008). The phytohormone ABA plays central roles in the tolerance of plants to cold and drought stress. Drought stress activates ABA-dependent and ABA-independent gene expression systems involving *ABA responsive element binding proteins* (*AREBs*), *MYB transcription factors* (*MYB*), *Drought responsive element binding 2* (*DREB2*), and *NAC* (*NAM*, *ATAF1*, *2* and *CUC*) transcription factors (TFs), while cold stress regulates ABA-independent pathway through *DREB1* TFs (Agarwal and Jha, 2010; Roychoudhury et al., 2013; Nakashima et al., 2014). These major TFs facilitate stress signaling and show differential transcript regulation in response to various stresses, and their overexpression resulted in up-regulation of a large number of genes directly or indirectly linked with stress tolerance in plants (Agarwal and Jha, 2010; Nakashima et al., 2014; Joshi et al., 2016).

The ubiquitin 26S proteasome system (UPS) has been shown to act as a critical mediator in plant response and adaptation to various abiotic stresses such as drought, cold and salinity (Stone, 2014). The UPS functions within the nucleus and cytoplasm to affect the accumulation of a wide range of stress-responsive proteins and degrade misfolded proteins that may be induced by abiotic stress treatments (Lyzenga and Stone, 2012). Ubiquitin-dependent proteasomal degradation contains multiple steps requiring the sequential action of three enzymes, namely ubiquitin activating enzyme (E1), ubiquitin conjugating enzyme (E2), and ubiquitin ligase (E3) (Stone, 2014; Sharma et al., 2016). Thousands of ubiquitination-related proteins are predicted in the *Arabidopsis* genome, which includes four 2 E1s, 37 E2s, and over 1300 E3 ligases (Kraft et al., 2005). E3s can be further divided into three major groups according to the conserved domain contained, including U-box, Really Interesting New Gene (RING) and Homology to E6-Associated Carboxyl-Terminus (HECT) groups (Stone, 2014). In plant, an increasing number of E2 and E3 encoding genes have been shown to be involved in abiotic stress responses. For example, transcript levels of *Arabidopsis ICE1* (*Inducer of CBF expression 1*), a basic helix-loop-helix (bHLH) transcription factor, are up-regulated in response to cold stress. ICE1 activity is inhibited by a RING-type E3 ligase, High Expression of Osmotically Responsive Gene 1 (HOS1), which is capable of catalyzing ICE1 ubiquitination *in vitro* and *in vivo*. Overexpression of *HOS1* increases sensitivity to freezing conditions of transgenic plants (Dong et al., 2006). In this case, ubiquitination of the regulatory protein would function as a negative regulator. On the contrary, UPS degrading negative players would activate the signaling pathways that are required for tolerance to stress (Stone, 2014). An example is R2R3-type MYB TF, *Botrytis Susceptible 1* (*BOS1*), which plays an important role in plants response to abiotic stresses (Luo et al., 2010). BOS1 can be degraded by RING-type E3 ligase BOI (BOS1 Interactor), which is involved in attaching the ubiquitins to BOS1 (Luo et al., 2010). This research suggests that UPS functions as a positive regulator in response to stress stimulus.

Cassava is an important tropical root crop worldwide and is an essential source of bio-fuel and starch for human consumption and animal feed. Although cassava can effectively utilize light, heat and water resources, cold and drought stresses cause great damage to the production of cassava. Improving the tolerance of cassava to cold and drought is an important strategy to increase root starch yield and supply. Recently, in cassava, many cold or drought stress-inducible genes acting in calcium and reactive oxygen species (ROS) signaling transduction pathways have been identified. In plant, stress-activated Ca^2+^ signaling transduction pathway is activated by Ca^2+^ spike from the cytoplasm for overcoming the cold damage and maintaining a balance between the intracellular and extracellular ionic concentrations (An et al., 2012; Zhu et al., 2013). the ROS signaling pathway that plays an important role in ROS detoxification, and hormone signaling pathway that are critical for the regulation of the cold- and/or drought-responsive transcriptome (An et al., 2012; Fu et al., 2016; Liao et al., 2016a). However, most studies have dealt with only one kind of stress, and little is known about how cassava plant integrates multiple signaling pathways, which may crosstalk or diverge at various points and form complex networks in response to both cold and drought stresses. Luckily, transcriptome profiling has been developed as a high-efficiency and extensive used approach to systematically investigate the gene expression in response to a diverse range of stresses globally across distant species (Chen et al., 2010; Zhu et al., 2013; Xie et al., 2015; Zheng et al., 2015; Muthusamy et al., 2016; Zhao et al., 2016). Additionally, transcriptomic data can provide much information on the metabolite, phytohormone, and transcript networks that control plant responses to various environmental stimuli. In this study, we analyzed the transcriptome of cassava seedlings exposed to cold and drought stresses and were able to identify various stress-responsive genes and related crosstalk networks involved in signal perception, transduction, and tolerance.

## Results

### Transcriptome Overview and Novel Transcript Identification

We analyzed our strand-specific RNA sequencing data of cassava shoot apices and leaves exposed to cold, drought stress and control conditions, respectively (Li et al., 2017). A total of ∼2.6 million RNA-Seq clean reads were obtained. After mapping the reads to the cassava genome using TopHat2, 74665, 78455, and 75088 transcript fragments were assembled using Cufflink on the samples under cold, drought stress and control conditions, respectively. By comparing these transcripts with the annotated genes in the cassava genome, we found that approximately 63% of total transcripts matched the predicted cassava genes and 33,033 transcripts were categorized as unique genes (**Table 1**). Notably, after filtering out low-abundance genes (FPKM < 1) and non-coding RNAs, we found a total of 481 protein-coding transcripts (1.46%) that showed no overlap with any annotated genes, and which were then identified as reference-dependent novel protein-coding genes (**Table 1** and Supplementary Table S1).

**Table 1:** Identification of novel protein-coding transcripts.

Number of transcripts	CK	Cold	Drought	Unique genes
Total assemble transcripts	74,665	78,455	75,088	33,514
Known protein-coding transcripts	46,842	49,656	47,244	33,033
Novel protein-coding transcripts	494	437	471	481


### Identification of Differentially Expressed Genes under Cold and/or Drought Stresses

**FIGURE 1:**
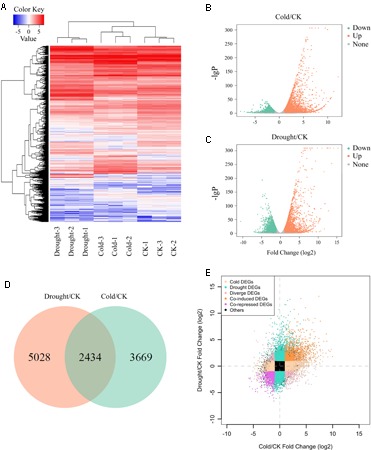
Variation in gene expression under cold or drought stress. **(A)** The heatmap of the fold change values in the RNA-seq data, and was used to visualize the gene expression pattern under cold and drought stress. **(B,C)** Volcano plot indicates the significance of DEGs between cold stress and control **(B)**, and between drought stress and control conditions **(C)**, respectively. **(D)** Venn diagram represents the overlapping DEGs number between cold- and drought-responsive genes. **(E)** Scatter plot indicates genes that were co-induced, co-repressed and specifically regulated by cold and/or drought treatment.

The expression level of 33033 annotated cassava protein-coding genes for each treatment (control check (CK), cold or drought) was assessed by counting the number of reads from the respective library that matched each contig, and normalizing to FPKM (fragments per kilo base of transcript per million mapped reads) to correct for the differences in sequencing depth for each dataset and gene length differences (**Figure 1A**). To determine data reproducibility, principal component analysis (PCA) was performed on the correlation of read count of annotated genes among samples. We found that the three replicates with the same treatment were clustered together, and were separated from samples with other treatments, indicating high reproducibility among biological replicates (**Supplementary Figure S1**). By comparing three RNA-seq libraries, CK, cold and drought, a plenty of differentially expressed genes (DEGs) were identified. To characterize the genes that were associated with cold and/or drought stress, we analyzed the most differentially regulated transcripts with a log2 ratio ≥ 1 or ≤-1 and FDR (false discover rate) ≤ 0.05. Using these standards, 6103 and 7462 genes were identified to be differentially expressed under cold and drought stress, respectively (**Figures 1B,C** and Supplementary Table S2). Under the cold treatment, 3547 genes were identified to be up-regulated and 2556 were down-regulated (**Figure 1B**), whereas under the drought treatment 3525 genes were up-regulated and 3937 genes were down-regulated (**Figure 1C**). Notably, a total of 2434 genes were differentially expressed under both cold and drought stress treatments, of which 1130 were co-induced and 841 were co-repressed by cold and drought (**Figures 1D,E**).

### Hormone Signaling Pathway in Response to Cold and Drought Stresses

According to previous research, both cold and drought stresses caused visible and similar morphological changes, including severe leaf dehydration and apical buds wilting. In addition, a clear impact on metabolites concentration was also observed in leaves of cold- and drought-stressed cassava plants (An et al., 2012). For instance, the concentrations of proline, total soluble sugars and malondialdehyde (MDA) increased after cold or drought treatment (An et al., 2012; Fu et al., 2016). Therefore, the similar physiological and molecular changes of cassava plant response to both stresses suggest the existence of crosstalk between the drought and cold stress signal perception and transduction pathways.

To explore the common and specific responses of cassava leaves to cold and drought stress, Gene Ontology (GO) analyses of all the DEGs were employed, and totally 111 significantly enriched gene categories in response to cold and/or drought stress were identified. The functions of these DEGs were categorized according to biological processes, including response to abiotic stimulus, response to lipid, cell communication, signal transduction and response to ABA stimulus pathways (Supplementary Table S3). Notably, a large number of DEGs were involved in hormone metabolic and signaling transduction pathways. Plant hormones such as auxin, ABA, jasmonic acid (JA), salicylic acid (SA), ethylene (ET), brassinosteroid (BR) and other small molecules play critical roles in regulating plant growth, nutrient allocation and stress tolerance to promote survival and acclimatize to varying environments throughout the life-span of plants (Fahad et al., 2015). In this study, six cold-specific signaling pathways and six drought-specific response pathways were identified. In the cold stress response, DEGs involved in ethylene and SA mediated signaling pathways were significantly enriched. While the “signaling pathway of auxin mediated” were identified in the drought stress response. Notably, two common hormone-related signaling pathways of DEGs that responded to both cold and drought stresses were identified: “response to ABA stimulus” and “response to JA stimulus pathways” (**Table 2**). Interestingly, we found that ABA-mediated signaling transduction pathway was regulated by cold stress, whereas drought stress mainly affected ABA biosynthetic and metabolic process. In plants, ABA signals are perceived by cellular receptors and a concept of activation of specific cellular ABA responses by perception in the distinct cellular compartments is currently emerging (Golldack et al., 2014). In that study, a total of 34 DEGs were identified as members of the ABA signaling pathway, which were mainly related to ABA transport, binding, signaling perception, transduction and ABA-dependent gene transcription, and included ATP-binding cassette (ABC)-containing transporter proteins, *PYRABACTINRESISTANCE-LIKE 2* (*PYL2*), *type 2C protein* ph*osphatases* (*PP2Cs*), *SNF1-RELATED PROTEINKINASEs* (*SnRK2s*) (Supplementary Table S4). Furthermore, *CORONATINE INSENSITIVE 1* (*COI1*) and *jasmonic acid ZIM-domain protein 2/9* (*JAZ2/9*), which function as JA receptors, were also identified response to both cold and drought stresses. It was reported that JAZs act as a key negative regulators of JA signaling in response to various abiotic stresses through repressing the activities of a plethora of TFs, such as bHLHs and MYBs (Goossens et al., 2016). The differentially expression of *JAZ2* and *JAZ9* indicated that JA signaling transduction related proteins were involved in response to cold and drought stresses in cassava.

**Table 2:** Gene ontology of biological process classification of DEGs under cold and/or drought stress.

GO biology process	GO ID	Cold condition Up/Down-regulated DEGs		Drought condition Up/Down-regulated DEGs		FDR
		Up	Down	Up	Down	
**Common**		
Response to stress	GO:0006950	534	292	572	402	2.47E-10
Response to stimulus	GO:0050896	890	542	913	846	3.40E-18
Response to abiotic stimulus	GO:0009628	270	152	323	240	0.0008
Response to hormone stimulus	GO:0009725	372	194	381	349	4.51E-14
Hormone-mediated signaling pathway	GO:0009755	278	147	259	263	9.07E-14
Signal transduction	GO:0007165	365	215	339	352	3.06E-09
Response to abscisic acid stimulus	GO:0009737	97	51	147	68	0.0087
Response to jasmonic acid stimulus	GO:0009753	43	12	49	18	0.0178
**Cold-specific**		
Ethylene mediated signaling pathway	GO:0009873	81	21	0	0	2.44E-08
Cellular response to ethylene stimulus	GO:0071369	81	22	0	0	7.29E-08
Response to salicylic acid stimulus	GO:0009751	48	17	0	0	0.0035
Cellular response to abscisic acid stimulus	GO:0071215	55	36	0	0	0.0008
Abscisic acid mediated signaling pathway	GO:0009738	43	32	0	0	0.0496
Response to heat	GO:0009408	42	25	0	0	0.0172
**Drought-specific**		
Response to auxin stimulus	GO:0009733	0	0	59	118	0.0003
Cellular response to auxin stimulus	GO:0071365	0	0	40	91	5.67E-06
Auxin mediated signaling pathway	GO:0009734	0	0	38	90	2.68E-06
Abscisic acid metabolic process	GO:0009687	0	0	18	3	0.0043
Abscisic acid biosynthetic process	GO:0009688	0	0	12	3	0.0194
Response to osmotic stress	GO:0006970	0	0	115	52	0.0453


A Kyoto encyclopedia of genes and genomes (KEGG) pathway enrichment analysis was performed to further investigate the changes in the transcriptome profiles between the drought and cold stress responses. Compared to the CK condition, DEGs involved in signaling transduction and metabolic pathways were significantly enriched in both cold and drought stress responses. **Table 3** presents the overall response pathways of cassava plants to cold and drought stress, respectively. One common feature was observed by pairwise comparison of both stress responses: plant hormone signal transduction pathways were notably enriched under the two stresses. A few differences between the two stress responses were also observed: (1) the drought-specific pathways were much more enriched than that of cold stress. Meanwhile, the number of DEGs whose expression was repressed by drought was higher than that of cold stress; (2) more metabolism- and photosynthesis-related DEGs were enriched after drought treatment. Taken together, these results pointed, for the first time, to relevant biological processes and pathways enriched as a consequence of the cold and drought stress treatments as compared to the control condition, and also indicated that both ABA and JA were involved in mediating these two stress responses.

**Table 3:** The significantly enriched pathways of DEGs under cold and drought stress.

Pathways	Map ID	Up DEGs	Down DEGs	*q*-value
**The significantly enriched pathways of DEGs under cold stress condition**		
Plant hormone signal transduction	map04075	45	32	3.11E-06
Plant-pathogen interaction	map04626	50	20	1.92E-05
Chemical carcinogenesis	map05204	10	9	0.001505
Drug metabolism – cytochrome P450	map00982	11	10	0.0016042
Metabolism of xenobiotics by cytochrome P450	map00980	10	9	0.0034902
Thiamine metabolism	map00730	8	0	0.0136633
**The significantly enriched pathways of DEGs under drought stress condition**		
Plant hormone signal transduction	map04075	56	53	1.745E-08
Biosynthesis of secondary metabolites	map01110	121	123	0.000152
Systemic lupus erythematosus	map05322	1	22	0.0009296
Metabolic pathways	map01100	195	226	0.0009296
Carotenoid biosynthesis	map00906	13	4	0.0013128
Fatty acid metabolism	map00071	15	6	0.0048858
Bisphenol degradation	map00363	8	7	0.006633
Limonene and pinene degradation	map00903	10	8	0.0121135
Polycyclic aromatic hydrocarbon degradation	map00624	8	7	0.023248
Alcoholism	map05034	3	20	0.0292773
Stilbenoid, diarylheptanoid and gingerol biosynthesis	map00945	9	8	0.0292773
Alpha-Linolenic acid metabolism	map00592	14	3	0.0501693
Photosynthesis – antenna proteins	map00196	0	6	0.0501693


### Characterization of Cold and Drought Stress Co-responsive DEGs

**FIGURE 2:**
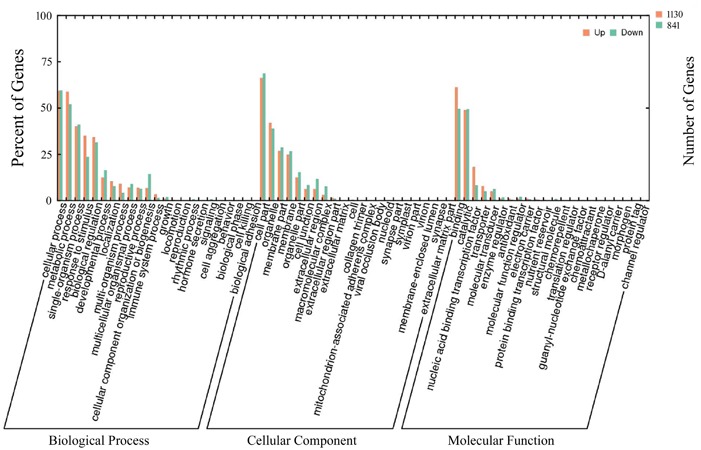
Gene ontology enrichment of stress co-responsive DEGs. The results are summarized into three main categories: biological processes, cellular components, and molecular functions. The *x*-axis corresponds to GO terms, and the *y*-axis shows the number of up- or down-regulated DEGs. A total of 1971 DEGs co-regulated by cold and drought stress were assigned to GO terms.

To gain further insight into whether similar trends existed in the transcriptomic profiles of cassava plants in response to cold and drought stresses, we analyzed the 1971 DEGs up-regulated or down-regulated by the two stress treatments (Supplementary Table S4). To classify these DEGs functionally, the GO database was utilized. In total, 46 GO terms were enriched and distributed in three categories, including biological process, cellular component and molecular function. Under the biological process category, cellular process (58.5% up-regulated, 57.6% down-regulated) was the largest group, followed by metabolic process (56.9% up-regulated, 52.8% down-regulated), response to stimulus (33.8% up-regulated, 23.2% down-regulated) and biological regulation (32.7% up-regulated, 30.5% down-regulated). In relation to the cellular component category, the DEGs associated with cell part (67.5% up-regulated, 70.1% down-regulated) and organelle part (39.2% up-regulated, 37.1% down-regulated), membrane part (25.9% up-regulated, 26.5% down-regulated) represented the most abundant categories. Among the top 3 GO terms enriched in the molecular function category, binding (61.9% up-regulated, 49.2% down-regulated), catalytic activity (48.3% up-regulated, 49.4% down-regulated) and nucleic acid binding transcription factor (15.8% up-regulated, 7.7% down-regulated) were accumulated more than the other terms (**Figure 2**). In addition, all of the above DEGs were annotated by KEGG pathway analysis, and the most enriched pathway was “biosynthesis of secondary metabolites,” followed by “metabolic pathways” and “microbial metabolism in diverse environments” (Supplementary Table S5). The analysis of the KEGG results suggested that both cold and drought stresses had strong effects on the biosynthesis of secondary metabolites.

### Identification of Stress-Related Protein Kinase Genes

**FIGURE 3:**
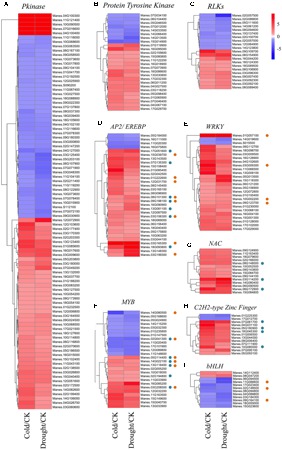
Expression pattern of cassava protein kinases and TFs in response to cold and drought stress. **(A–I)** Heatmaps were generated from the fold change values of *Pkinase* **(A)**, *Protein Tyrosine Kinase* **(B)**, *RLKs* **(C)**, *AP2/ERFBP* **(D)**, *WRKY* **(E)**, *MYB* **(F)**, *NAC* **(G)**, *C2H2-type Zinc Finger* **(H)**, and *bHLH* **(I)** family numbers under cold and drought stress as compared to the control condition. The blue and orange spots indicated DEGs responding to ABA and JA signaling, respectively.

According to the “molecular function” GO terms in **Figure 2**, catalytic activity and transcription factor activity were the two major categories. Therefore, proteins with catalytic function act as master regulators involved in stress signal perception by modulating downstream proteins activities. In the past decades, plant protein kinases have been found to be conserved components of signaling networks such as the perception of plant hormones and various adverse environmental conditions. We identified a wide range of protein kinase family members in the RNA-seq data, mainly composed of 91 pkinase, 26 protein tyrosine kinase and 21 receptor-like protein kinases (RLKs) family members according to the domain classification in the Pfam database (Finn et al., 2014; **Figures 3A–C** and Supplementary Table S6). Among pkinase and protein tyrosine kinase families, a number of common stress-related DEGs that responded to both cold and drought stresses were identified: 4 MAPK family members and 12 CDPK family members. The majority of these DEGs were up-regulated by both cold and drought stresses, suggesting they may play positive roles in stress tolerance. RLKs are a family of transmembrane receptors with an intracellular serine/threonine kinase domain and perform critical functions in modulating diverse biological processes by perceiving extracellular stimuli and activating downstream signaling responses (Ye et al., 2016). In both the cold and drought stress responses, ten *Leucine-Rich Repeat Receptor-Like protein Kinases* (*LRR-RLKs*), seven *Malectin* or *Malectin-like domain-containing Receptor-Like Kinases* (*MRLKs*) and four *Legume lectin Receptor-Like Kinases* (*LecRLKs*) were identified. Among 21 *RLK* family members, a total of 11 *RLKs* were significantly induced by the two stresses, especially after cold treatment, while the other 10 exhibited decreased expression patterns following all stress conditions (**Figure 3C**). The diverse expression of *RLKs* indicated that early cold and drought stress signaling perception and transduction may be enhanced.

### Identification of Transcription Factors in Responses to Cold and Drought Stress

Transcription factors play an essential role in plant growth and stress responses (Agarwal and Jha, 2010). We investigated the biological functions of TFs that responded both to cold and drought stress in cassava. A total of 211 co-responsive DEGs were identified as TFs, which belonged to 42 gene families based on Pfam database (Finn et al., 2014). The number of stress-induced TFs (125) was much larger than the number of stress-suppressed ones (47), suggesting that transcriptional activation may be dominant over repression (Supplementary Table S6). Among these TFs, the most abundant transcription factor family was *APETALA 2*/*Ethylene-Responsive Element Binding Factor* (*AP2/EREBP*, number of DEGs: 32), followed by *WRKY* (26), *MYB* (26), *NAC* (15), *Zinc-Finger* C2H2 (13) and *bHLH* (12) family. The highest number of TFs was induced by the two stresses, except for the *bHLH* family (**Figures 3D–I**). Notably, 20 and 21 TFs were identified to be ABA- and JA-responsive according to GO analysis, respectively (**Figure 3**).

The *AP2/EREBP* family was originally identified as key regulators in the manifestation of the stress response in plants, as evidenced by many well-characterized *DREBs*/*CBFs*, which belongs to DREB subfamily of *AP2* TFs (Mizoi et al., 2012). In our study, all members of *AP2*/*EREBP* family are classified into four subgroups, including AP2, RAV, ERF and DREB subgroups. For example, Manes.12G031700, Manes.15G039700 and Manes.03G165100 encode members of ERF subgroup (Supplementary Table S6), which is known to be involved in various biotic stress responses (Cheng et al., 2013; Zhu et al., 2014). In many studies TFs defined by the WRKY domain, a highly conserved 60 residues long DNA binding domain (Rushton et al., 2010). Many have been shown *WRKYs* to be involved in plant responses to abiotic stresses (Rushton et al., 2010; Chen et al., 2012). Our transcriptome analysis showed that most of the differentially expressed *WRKYs* (92.3%) were induced by cold and drought treatment (**Figure 3E**). The *MYB*s are widely distributed in plants, forming a large family characterized by conserved MYB domain, and almost all *MYBs* are stress- or hormone-responsive (Yanhui et al., 2006; Wang et al., 2016). In our study, 26 *MYB* or *MYB*-related family genes were responsive to cold and drought, with 14 stresses-elevated and 12 stresses-repressed (**Figure 3F**). The number of up- and down-regulated MYBs was nearly equal, suggesting a diverse range of functions for this TF family in response to cold and drought stress. The *NAC* family has been demonstrated to be associated with diverse biological processes and responding to many biotic and abiotic stresses in plant (Nuruzzaman et al., 2013). We found all of the 15 co-responsive *NAC* genes identified in this study were up-regulated after stress treatments (**Figure 3G**), suggesting that these *NAC* genes may play a positive role in regulating stress-associated genes. The Cys2/His2 (C2H2) zinc finger protein belongs to *Zinc-Finger Protein* (*ZFP*) family that play various roles in the plant stress response (Ciftci-Yilmaz and Mittler, 2008). In *Arabidopsis*, expression of Cys2/His2-type ZFPs *AZF1* and *STZ* are induced by abiotic stresses and ABA treatment. Experiments indicated that they function as positive regulators in stress response (Sakamoto et al., 2004). Members of the *bHLH* gene family encode TFs and play diverse functions in plant growth and development, such as trichome differentiation, seed development, stamen development, shoot and inflorescence branching (Schmitz and Theres, 2005; Balkunde et al., 2010; Sun et al., 2010). It is worth noting that the majority of *bHLH* (83.3%) family members were down-regulated based on the RNA-seq data (**Figure 3I**), suggesting that cold and drought stresses may conserve energy by inhibiting plant growth, and adapt to the environmental stimuli.

In addition to the above-mentioned TFs, we also found four TFs families, including *Heat Stress transcription Factors* (*HSFs*, number of DEGs: 10), *GRAS* (9), *Homeobox* (8), and *Zinc-Finger Dof* (7), were cold-and drought-responsive in our study (Supplementary Table S6). Most of these TFs showed the inducible expression pattern after cold and drought treatments. Taken together, the large number and diverse expression patterns of co-responsive TFs indicated their involvement in the complex signaling networks of the two stresses responses.

### Identification of DEGs Associated with Protein Degradation

By modulating the activity and accumulation of functional proteins, the UPS acts as key regulator in mediating growth and abiotic stress response of plants. Recent reports in this field have identified numerous ubiquitin-conjugating (E2) enzymes and ubiquitin ligases (E3) exhibiting altered expression levels during abiotic stresses (Lyzenga and Stone, 2012; Stone, 2014). In this study, a great number of degradation-associated proteins were involved in response to both cold and drought stress. Among these DEGs, all of three E2 enzymes were found to be up-regulated under stresses. Meanwhile, a total of 29 E3s composed of 16 RING-type, 12 U-box-type and 1 HECT-type members were also identified and co-regulated by the two stresses (**Table 4**). As reported in previous studies, E2s and E3s are involved in a range of protective responses that help plants to against various stresses (Stone, 2014). The identification of E3 ubiquitin ligases, in our study, establishes a direct link between the UPS and plant stress tolerance.

**Table 4:** Differentially expressed genes involved in ubiquitin-dependent protein catabolic pathway.

Gene ID	Fold change		Pfam ID	Pfam description
	Cold	Drought		
**Ubiquitin-conjugating enzymes (E2)**		
Manes.05G154600	2.51	3.78	pfam00179	E2 enzyme UBC16
Manes.07G104200	2.06	2.80	pfam00179	E2 enzyme UBC11
Manes.03G110600	2.23	4.83	pfam00179	E2 enzyme UBC25
**RING-type ubiquitin-protein ligases (E3)**		
Manes.13G147200	3.52	2.48	pfam13923	C3HC4 type Ring finger E3
Manes.14G016800	2.14	3.08	pfam13920	C3HC4 type Ring finger E3
Manes.06G172200	2.25	2.79	pfam13920	C3HC4 type Ring finger E3
Manes.11G144400	2.04	2.05	pfam13920	C3HC4 type Ring finger E3
Manes.05G145000	0.33	0.08	pfam13920	C3HC4 type Ring finger E3
Manes.15G008300	0.24	0.33	pfam13920	C3HC4 type Ring finger E3
Manes.07G063200	2.66	2.14	pfam13639	Ring finger E3
Manes.08G113500	2.59	2.24	pfam13639	Ring finger E3
Manes.08G092000	2.64	6.15	pfam13639	Ring finger E3
Manes.03G055000	5.06	2.34	pfam13639	Ring finger E3
Manes.18G079700	3.51	4.26	pfam13639	Ring finger E3
Manes.08G123000	2.57	3.42	pfam13639	Ring finger E3
Manes.14G058200	6.97	17.32	pfam13639	Ring finger E3
Manes.13G097800	7.27	10.40	pfam13639	Ring finger E3
Manes.09G164200	5.41	3.65	pfam13639	Ring finger E3
Manes.16G051700	0.20	0.26	pfam13639	Ring finger E3
**U-box-type ubiquitin-protein ligases (E3)**		
Manes.02G176900	6.56	32.52	pfam04564	U-box domain E3
Manes.16G113200	4.20	23.97	pfam04564	U-box domain E3
Manes.17G086300	8.94	30.12	pfam04564	U-box domain E3
Manes.08G034000	8.72	4.72	pfam04564	U-box domain E3
Manes.18G089700	54.62	7.66	pfam04564	U-box domain E3
Manes.17G031300	3.29	3.18	pfam04564	U-box domain E3
Manes.01G121300	68.07	3.22	pfam04564	U-box domain E3
Manes.12G029900	2.30	36.39	pfam04564	U-box domain E3
Manes.09G046000	8.50	13.65	pfam04564	U-box domain E3
Manes.14G172900	14.78	9.08	pfam04564	U-box domain E3
Manes.06G103300	2.63	126.52	pfam04564	U-box domain E3
Manes.17G075700	0.43	0.38	pfam04564	U-box domain E3
**HECT-type ubiquitin-protein ligase (E3)**		
Manes.02G091100	0.3129428	0.3354057	pfam00415	HERC2


### Identifying the Interaction Network of Co-Responsive DEGs

**FIGURE 4:**
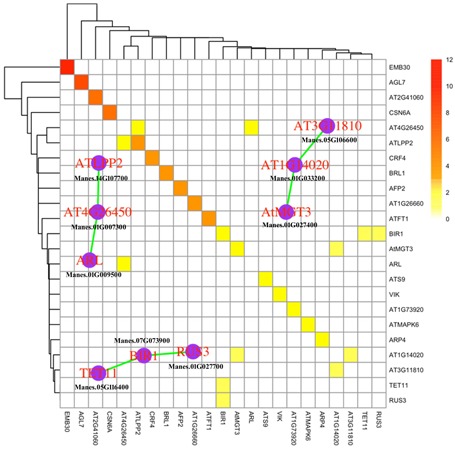
Protein–protein Interaction network of cassava co-responsive DEGs homologs in *Arabidopsis* that function in hormone signal, protein kinase, protein degradation and TFs. PPI database were downloaded from BioGRID (Stark et al., 2006) and *Arabidopsis* interactome map (*Arabidopsis* Interactome Mapping Consortium, 2011). The color of heatmap indicates number of evidences for PPI. The signal in diagonal line indicates self-interaction. The graph with purple vertex and green line highlights the PPI network of cassava homologous genes in *Arabidopsis*.

In recent years, it has been demonstrated that the computational analysis of protein–protein interaction (PPI) networks is becoming increasingly useful to identify the biological processes and signaling pathways (Natale et al., 2014). To investigate the potential PPI networks of co-responsive DEGs, we identified the homologs of DEGs in *Arabidopsis* that function in hormone signaling, protein kinase, protein degradation and TFs, and assembled putative networks based on experimentally validated interactions database *Arabidopsis* interactome map (*Arabidopsis* Interactome Mapping Consortium, 2011) and BioGRID (Stark et al., 2006). As a result, we found 23 proteins that can self-interact or interact with other members in the network. Among them, there are three interactive protein complex, including LPP2 (LIPID PHOSPHATE PHOSPHATASE 2), BIR1 (BAK1-INTERACTING RECEPTOR-LIKE KINASE 1), and MGT3 (MAGNESIUM TRANSPORTER 3) (**Figure 4**). AtLPP2 negatively regulates the activity of ABI4, an AP2-type TF, in response to ABA signals (Katagiri et al., 2005). The proteins from these networks were activated, indicating an increase in the ABA response in cassava leaves under stress treatments, which is consistent with our results in **Table 2**. Previous work showed that BIR1 functions as a negative regulator of plant immunity through regulating multiple plant resistance signaling pathways (Liu et al., 2016). MGT3 was involved in transporting magnesium, which acted as a key osmoticum maintaining growth in low calcium concentrations in plants (Conn et al., 2011). Together, the networks of protein complexes identified in this study were mainly involved in ABA signaling transduction, antimicrobial response and osmotic adjustment, and may represent key crosstalk nodes in the cold and drought stress responses in cassava.

### Validation by Real-Time RT-qPCR of Selected Stress-Responsive Genes as Detected by the RNA-Seq Data

**FIGURE 5:**
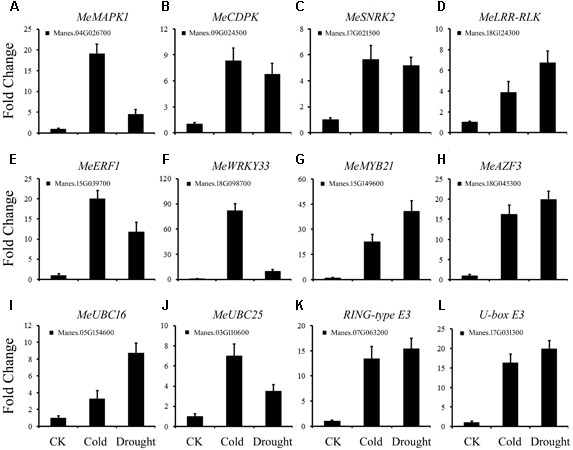
Confirmation of the expression patterns of DEGs using quantitative RT-PCR. **(A–L)** The expression pattern of DEGs under cold or drought stress. Data are presented as mean ± SD of three independent assays. *MeACTIN* was used as the reference gene.

To further study the cold and drought co-responsive DEGs listed in Supplementary Table S4 in cassava, 12 genes were analyzed by real-time quantitative PCR (qRT-PCR). The temporal expression patterns of the DEGs detected by qPCR correlated well with the RNA-seq data. Among the 12 DEGs, 4 protein kinases (Manes.04G026700, Manes.09G024500, Manes.17G021500, and Manes.18G124300) showed differential expression under cold and drought stress (**Figures 5A–D**). Four TFs *MeERF1* (**Figure 5E**), *MeWRKY33* (**Figure 5F**), *MeMYB21* (**Figure 5G**) and *MeAZF3* (**Figure 5H**) also showed strong induction by cold and drought treatments, except *MeWRKY33*, which exhibited significant variation between cold and drought-treated plants. Two E2s (*MeUBC16* and *MeUBC25*) and two E3s (Manes.07G063200 and Manes.17G031300) were also up-regulated after both cold and drought treatment (**Figures 5I–L**). The striking similarity in the response pattern between the two types of stresses suggested that these genes may play important roles in cassava. Thus, the functions of these genes in cassava will be further studied.

## Discussion

Cassava is an economically important crop grown for its starchy roots and its good adaptation to drought and heat stress conditions. Molecular mechanisms involved in cassava responses to diverse adverse environment stimuli have been studied using microarray chips and high-throughput sequencing (Lokko et al., 2007; An et al., 2012; Utsumi et al., 2012; Turyagyenda et al., 2013; Zhao et al., 2015; Fu et al., 2016). An increasing number of drought- or cold stress-responsive genes have been reported. However, most studies are focused on one kind of stress, no systematic consensus on genes that correspond to crosstalk network for cold and drought stress perception, signal transduction and tolerance of cassava has been reported. Identification of key regulators at the nodes and branches between cold and drought stress signaling pathways will provide insights into the mechanism involved these stress responses in cassava. Here, we focused on the common effects of both cold and drought stresses on the cassava plants response. In this research, we performed a comparative analysis of the transcriptome profiles of cassava leaves under CK, cold, and drought stress conditions, our results would provide fundamental insights into gene expression changes and signaling transduction networks of cassava in response to temperature and water changes.

### The Role of Hormones in Plant Response to Cold and Drought Stress

In response to stress, a diverse range of genes involved in various biochemical and physiological processes are involved, which can lead to adjustment of the cellular milieu and plant tolerance. In this study, we obtained the expression information of 33,033 protein-coding genes from cold- and drought-treated cassava plants. Using comparative analysis of stress-responsive genes from different conditions, 1971 genes were identified as commonly induced or repressed by cold and drought treatments. GO analysis revealed that DEGs associated with the hormonal signaling pathways, including ABA and JA signaling pathways, were significantly enriched.

The prominent role of ABA in plant response to abiotic stress conditions has long been studied. Under drought conditions, ABA is known to stimulate closure of stomata, resulting in maintenance of water balance and modulate plant growth through the regulation of stress-responsive genes (Verma et al., 2016). Previous studies have found that levels of endogenous ABA were increased under cold, salt and drought stress conditions in many plant species. Exogenous application of ABA to plants can elevate their tolerances to abiotic stresses (Sah et al., 2016). In our research, a large number of DEGs of “response to ABA stimulus” were significantly up- or down-regulated by cold and drought stress, such as ABC-containing transporter protein (*MeABCG25*, Manes.18G101300), ABA receptor *PYL2* (Manes.16G079500), *PP2Cs* (Manes.18G136400, Manes.07G131300, Manes.10G010600, and Manes.03G036900), *SnRK2* (Manes.17G021500), and TFs (*MYBs*, *NACs* and *Zinc-finger C2H2s*). In *Arabidopsis*, ABC transporter protein *AtABCG25*, is required for exporting ABA from vascular tissues (Sah et al., 2016). AtPYL2 acts as an ABA receptor, which can bind ABA directly (Yin et al., 2009). After binding to ABA, this receptor can suppress the phosphatase activity of PP2Cs, the negative regulator of *SnRK2* (Park et al., 2009). However, a common feature of PP2Cs is that their expression is induced by high ABA levels and abiotic stresses (Yang et al., 2017). In rice, the overexpression of *OsPP108*, which encodes a PP2C family protein, confers ABA insensitivity and tolerance to stresses, such as high salt and drought (Singh et al., 2015). The SnRK2 family of protein kinases plays a central role in cellular responses to abiotic stresses and mediates ABA signaling by phosphorylating their substrate proteins such as TFs, metabolic enzymes, and ion channels (Sah et al., 2016). In cassava, the significantly stress-induced expression pattern of *MeABCG25*, *PP2Cs*, and *SnRK*2 suggested that these TFs play regulatory roles in the perception and transduction of ABA signals under stress conditions.

Recent studies have highlighted that JA plays an important role in the regulation of abiotic stress tolerance under osmotic stress conditions (Ahmad et al., 2016). It has been demonstrated that JAs regulate the gene expression involved in stress responses though up-regulating expression of antioxidant enzymes and eliciting plant secondary metabolism (Ahmad et al., 2016). We found that 50 DEGs associated with “response to JA stimulus” signaling pathways were significantly enriched under both cold and drought stress. JA-dependent signaling is initiated by the interaction of JA with the receptor COI1 which causes conformation changes to interact with bHLH TFs. This interaction results in ubiquitination of JAZs by the Skp1/Cullin/F-box (SCF) complex, and releases bHLH TFs suppression, triggering downstream JA responses (Goossens et al., 2016). As previously reported (Valenzuela et al., 2016), COI1, which act as an E3 ubiquitin ligase, was significantly induced under cold and drought treatments. However, two JAZ genes, JAZ2 and JAZ9, the key negative regulator of JA signaling pathway, also significantly increased by stresses, consistent with the results of previously reported studies (Huang et al., 2016; Valenzuela et al., 2016), suggesting possible feedback mechanisms triggered by high levels of JA accumulation. Taken all together, the exploration of ABA and JA signaling molecules can be beneficial to examine the details of underlying molecular mechanisms of these hormone signals transduction, and much work need to be done in near future in cassava.

### Signal Transduction in Plant Response to Cold and Drought Stress

In total, we identified 1130 induced and 841 repressed DEGs. Molecular functional classification of the these genes shows a large number of genes that are predicted to be involved in signal transduction, transcription regulation, secondary metabolism, transport facilitation and protein degradation. As expected, protein kinases and TFs corresponded to the most regulated genes, suggesting that cold and drought stress signal transduction pathways overlap at several points. The important Receptor-like Kinases (RLK) group which includes members like *LRR-RLK*, *MRLK*, and *LecRLK*, have been previously shown to be involved in mediating the cellular response to various environmental cues, hormonal signals and stress perception (Ye et al., 2016). Many studies have focused on the characterization and function of RLKs in abiotic stress tolerance, specifically for drought, cold and salinity. In rice, *LRR-RLK* gene FON1 increased drought tolerance of transgenic rice plants through phosphorylating key components of the ABA signaling pathway and activating the ABA signal (Feng et al., 2014; Ye et al., 2016). Another well studied example is *CRLK1*, a calcium-regulated *RLK*, which modulated cold tolerance through phosphorylating MAP kinases in plants, and promoted expression of cold stress response genes, finally regulating plant adaptation to cold stress (Furuya et al., 2013; Ye et al., 2016).

In addition to the stress signal perception cascades, there is a second group of DEGs with protein kinase activity. It was reported that MAPKs and calcium-binding related kinases were central regulators in signal transduction, connecting the perception of external stimuli to cellular responses (Boudsocq and Sheen, 2013; Xie et al., 2015; de Zelicourt et al., 2016). In the study, a total of 4 *MAPKs*, 12 *CDPK*s and a large number of serine-threonine protein kinases were identified as commonly stress-responsive members. These kinases may not only function as signals transducers but also work as central regulators through phosphorylating stress-responsive proteins under cold and drought stress. In *Physcomitrella patens*, ARK encodes a MAP kinase kinase kinase (MAP3K), and acts as a novel regulatory component of ABA signaling transduction pathway via phosphorylating SnRK2s (Saruhashi et al., 2015). Many TFs are also functionally regulated by post-translational phosphorylation mechanisms. For instance, bZIP, NAC and DREB TFs are generally phosphorylated by the serine-threonine kinases, MYB/MYC and WRKY TFs are phosphorylated by MAPKs, and CDPKs are involved in phosphorylation of some bZIP TFs (Gahlaut et al., 2016).

### The Role of Transcription Factors in Plant Response to Cold and Drought Stress

It is known that TFs act as regulatory proteins in a synchronized manner by regulating a set of downstream genes under their control and consequently enhance the stress tolerance of the plant. A large number of TFs including *WRKYs*, *ERFs*, and *MYBs* have been shown to exhibit induced expression during exposure to cold or drought stress, suggesting that these genes may be used to improve stress tolerance in cassava (An et al., 2012; Turyagyenda et al., 2013; Fu et al., 2016). In our research, different families of TFs including up-regulated *AP2/ERFBP*, *WRKY*, *MYB*, *NAC*, *GRAS*, *C2H2 ZFP*, and down-regulated *bHLH* were identified in cassava response to cold and drought stress. Among these TFs, we identified a wide range of ABA-dependent TFs, including 7 *AP2/ERFBPs*, 6 *MYBs*, 3 *NACs*, and 4 *zinc-finger* TFs. In addition, 7 *AP2/ERFBP*s, 6 *MYBs*, *4 WRKYs*, and 3 *bHLHs* TFs were shown to be involved in the regulation of core JA signaling (**Figure 3**). There are many evidences of implication of *AP2/ERFBP* genes especially *DREBs* in drought, salt, heat and cold stress responses in plants (Mizoi et al., 2012). In *Arabidopsis*, DREB2s have been reported to promote the expression of downstream genes to enhance cold and drought tolerance in transgenic plant (Sakuma et al., 2002). Ectopic overexpression of *Arabidopsis DREB1* in cassava improves the plant performance against cold and drought stresses but represses plant growth, and reduces storage root yield (An et al., 2016). In previous studies, a total of 85 *WRKY* and 166 *MYB* family numbers in cassava have been identified, respectively, and their expression patterns under drought stress have also been characterized (Liao et al., 2016b; Wei et al., 2016). In our research, abiotic stress related gene like *WRKY22* (Manes.01G057100), *WRKY33* (Manes.18G098700), *MYB78* (Manes.03G208500), and MYB21 (Manes.15G149600) was found to accumulate after cold and drought stress treatment. Based on previous studies, *WRKY22* is considered to modulate the interplay between the SA and JA pathways in response to a wide range of biotic and abiotic stimuli (Kloth et al., 2016). WRKY33 is well known as a substrate of MPK3/MPK6 in reprogramming the expression of camalexin biosynthetic genes, which drives the metabolic flow to camalexin production in *Arabidopsis* challenged by biotic stress (Mao et al., 2011). Furthermore, genetic and physiological experiments demonstrate that the *R2R3-MYB* transcription factor *MYB21* functions as direct targets of JAZs to regulate male fertility specifically, indicating MYB TFs play an essential role in JA signaling transduction pathway (Song et al., 2011). Taken together, according to our results, a large number of TFs were involved in cold and drought stresses responses in a hormone-dependent manner, suggesting that TFs play an important role in modulating the two stresses signal transduction and may crosstalk at many steps.

### The Role of Ubiquitin-Mediated Degradation System in Plant Response to Cold and Drought Stresses

Recently, the involvement of the ubiquitin proteasome system (UPS) at the cellular level has received great attention. In plants, ubiquitin-mediated degradation plays an important role in growth, hormonal signaling, abiotic stress, embryogenesis, and senescence (Stone, 2014). The expression of three ubiquitin conjugating enzyme E2 (*UBC* E2) including *UBC11* (Manes.07G104200), *UBC16* (Manes.05G154600), and *UBC25* (Manes.03G110600) was found to increase after cold and drought stress treatments in cassava. Accumulating evidences show that the expression of *UBC* E2 genes from a number of plant species is regulated by development and also by environmental conditions (Xu et al., 2009). Overexpression of soybean *GmUBC2* resulted in improved drought tolerance in *Arabidopsis* (Zhou et al., 2010). Therefore cassava *UBC11/16/2*5 might play some role in the response to biotic stresses, given that they were up-regulated after stress treatment.

In addition to E2 enzymes, E3 ligases have also been extensively studied in development and in signaling responses during abiotic stress, and act as either negative or positive regulators in stress signal transduction (Lyzenga and Stone, 2012). We identified 13 RING type and 11 U-box type E3s were strongly induced by cold and drought stress, respectively. It was previously shown that overexpression of *Arabidopsis PUB22* and *PUB23*, which encode U-box E3 ligases, were hypersensitive to drought stress, indicating that PUB22 and PUB23 act as negative regulators in osmotic stress tolerance (Cho et al., 2008). By contrast, RING finger E3 ligases SDIR1 (SALT- AND DROUGHT-INDUCED RING FINGER1) act as positive regulators of ABA signaling. Overexpression of *SDIR1* leads to ABA hypersensitivity and ABA-associated phenotypes, such as salt hypersensitivity in germination, enhanced ABA-induced stomatal closing, and enhanced drought tolerance (Zhang et al., 2007). Therefore, positive regulation of RING type and U-box type E3s under cold and drought stress treatment in cassava suggest that E3s may play a role in the response to stress conditions through ubiquitination and the resulting degradation of essential components in these stress signaling pathways.

### Hierarchical View of Crosstalk between Cold and Drought Stress

**FIGURE 6:**
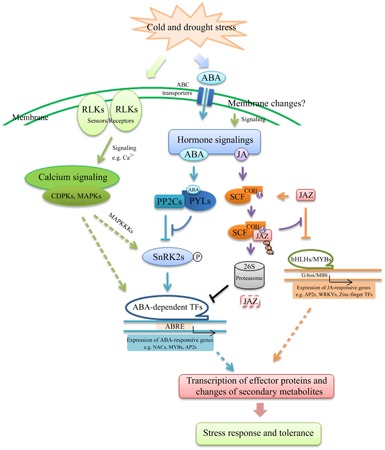
Models describing the signaling pathways involved in the acquisition of cold and drought tolerance.

Plant survival under multiple and simultaneous environmental conditions implies complex processes of signal reception, transmission, amplifications and interaction. As shown in **Figure 6**, signal transduction pathways triggered by cold and drought share a plenty of regulatory proteins that transduce the related signaling into downstream processes that result in resistance to such stresses. In general, the cold and drought stress signals are perceived by several receptors at the cell membrane, such as LRR-RLKs, MRLKs and LecRLKs, followed by calcium molecules transduction to activate downstream stress-responsive genes in response to abiotic stress. The change of second messengers stimulates downstream signals mediated by combinations of protein phosphorylation cascades, such as MAPKs and CDPKs. In addition, ABA and JA usually increase during early stages of stress. In the presence of ABA, PYL receptors bind to ABA and prevent PP2C from phosphorylating SnRK2. The SnRK2s are then phosphorylated by MAP3Ks and activated. The active SnRK2s can phosphorylate downstream TFs and activate the expression of ABA-dependent genes. The cold and drought response has been reported to involve both ABA-dependent and independent pathways (Roychoudhury et al., 2013). In the ABA-independent pathway, the transcription factor of GRAS and bHLH has also been implicated in dehydration stress signaling. On the other hand, in the ABA signaling pathway, TFs such as MYB, AP2 and NAC are believed to activate the transcription of specific target genes. Meanwhile, upon increase of JA/JA-Ile levels by cold and drought stress, SCF^COI1^ binds to JA and facilitates the degradation of JAZs protein, repressors of MYCs/MYBs, via the ubiquitin-26S proteasome pathway. Therefore, MYCs/MYBs can switch on transcription of JA -responsive genes. Therefore, ABA- and JA-induced signaling pathways might regulate transcription or directly/indirectly interact with cold and drought stress signaling networks. Moreover, we also obtained a large amount of information on ubiquitin-dependent protein catabolic processes, which was involved in cold and drought stress signaling by either positively or negatively regulating the accumulation of the stress-responsive proteins. Furthermore, we identified PPI network, in which the genes were responsive to both drought and cold, including three complexes of interacting proteins. Taken together, our study indicated that stress signal perception and transduction were critical components of the cold and drought stress responses in cassava plants. Meanwhile, these common signaling-related DEGs could be key nodes of complex networks in response to cold and drought stresses, and are candidates for further studies as potential targets to improve resistance to multiple stresses through genetic engineering.

## Conclusion

A comprehensive transcriptome-based characterization of cold and drought responsive DEGs was conducted in cassava for the first time. Our results revealed that the expression of 1130 genes was induced, and 841 genes were repressed in cassava following both cold and drought stress treatments. We identified important crosstalk and divergence of the signaling transduction pathways between these two stresses. First, the great majority of the DEGs were related to signal transduction, TFs and the ubiquitin-proteasome system. Second, hormone signaling, transcription activation/repression, and ubiquitin-dependent protein catabolic pathways were important response events in the mechanism underlying the crosstalk of the cold and drought response mechanisms of cassava. Our results revealed functional specialization of gene families when plant cells encountering different stresses. The results of this research provide a theoretical basis for further investigation of the molecular mechanisms involved in the cassava response to cold and drought stress, and also provide valuable information for future work.

## Materials and Methods

### Plant Material and Stress Treatment

Cassava (*Manihot esculenta*) cultivar (TMS60444) was used in the study. As previously described, the stems about 1.5 cm in size with one bud were cut and planted in MS plate for 2 weeks in a growth chamber at 26 ± 2°C, with a photoperiod of 16 h light and 8 h dark (Li et al., 2017). For cold treatment, plants with a uniform growth status were treated at 4°C for 24h in a chamber under light (Li et al., 2017). The shoot apex and the youngest leaves were collected and then were frozen in liquid nitrogen for RNA extraction. For drought treatment, seedlings were planted in MS with 20% PEG6000 and harvested at 6 h after treatment (Li et al., 2017). In all cases, parallel and untreated plants at the same stage were used as controls. At least three replicates for each treatment were harvested (Li et al., 2017).

### RNA Isolation, DNA Synthesis, and Real-Time Quantitative PCR

Total RNA was extracted using the RNA Plant kit (OMEGA) according to the manufacturer’s instructions. The 1st strand cDNA were synthesized in a 20 μl reaction solution containing 2 μg total RNA samples of CK, as well as abiotic stressed plants using PrimeScriptTM RT reagent Kit (Takara). Real-time qPCR assays were performed using the SYBR Premix Ex TaqTM (Takara) followed the manuals from the manufacturers. A one-step RT-PCR procedure was performed in all experiments. For each sample, qRT-PCR reaction was repeated three times and the relative mRNA expression level was calculated as 2^-ΔΔCt^. The cassava *MeACTIN* gene was used as a constitutive reference. All the primers used were as listed in Supplementary Table S7.

### RNA-Seq and Data Analysis

For RNA-seq, cassava shoot apices and youngest leaves for each treatment were collected and pooled separately to prepare 3 cDNA libraries (CK- control treatment; cold- cold stressed; drought -drought stressed). As previously described (Li et al., 2017), A total of 140 gigabase in-depth sequencing of library was performed initially on a HiSeq 2500 instrument that generated paired-end reads with 125 nucleotides. After filter out the adapter and low quality sequences, TopHat2 (Trapnell et al., 2012) was used to map the clean reads to cassava genome. Then, reference genome-based transcriptome assembly was performed to generate non-redundant unigenes using Cufflinks v2 method (Trapnell et al., 2010). The expression levels of unigenes were calculated and then normalized to FPKM (fragments per kilo base of transcript per million mapped reads) (Wagner et al., 2012). The DEGs between treatment and control were calculated by cuffdiff program, and the significant DEGs were filtered with ratio more than two fold change and false discovery rate (FDR) less than 0.05. The RNA-seq data was submitted to Sequence Read Archive (SRA) in NCBI and the accession number is SRP101302.

### Functional Annotation and Classification

To predict the potential functions and biological pathways of the genes, we annotated the genes using the NR protein database (NCBI), GO (Young et al., 2010) and KEGG databases. As described previously, GO enrichment analysis of genes was implemented by using the GO seq R package. KOBAS software was used for testing the statistical enrichment of DEGs in KEGG pathways^1^ (Kanehisa et al., 2008).

### Protein–Protein Interaction Network

The PPI database were downloaded from *Arabidopsis* interactome map (*Arabidopsis* Interactome Mapping Consortium, 2011) and BioGRID (Stark et al., 2006). The *Arabidopsis* homologs of cassava DEGs were annotated, and number of PPI evidences for pairwise genes from PPI database was extracted. The PPI matrix with evidence number were presented as heatmap using R package “pheatmap,” and the graph network were displayed using R package “igraph.”

## Author Contributions

SL and MP participated in the design of the study. SL and XY analyzed the RNA-seq data. ZC, XLY, MBR, and WL carried out the molecular biology experiments. All authors read and approved the final manuscript.

## Conflict of Interest Statement

The authors declare that the research was conducted in the absence of any commercial or financial relationships that could be construed as a potential conflict of interest.

## Footnotes

1 http://www.genome.ad.jp/kegg/

## Supplementary Material

FIGURE S1 Principal component analysis of three samples for each treatment.
