# Supplementary figures and images for: Global Gene Expression Analysis Reveals Crosstalk between Response Mechanisms to Cold and Drought Stresses in Cassava Seedlings
Source: Front Plant Sci. 2017 Jul 18;8:1259. doi: 10.3389/fpls.2017.01259 (PMC5513928; doi:10.3389/fpls.2017.01259)

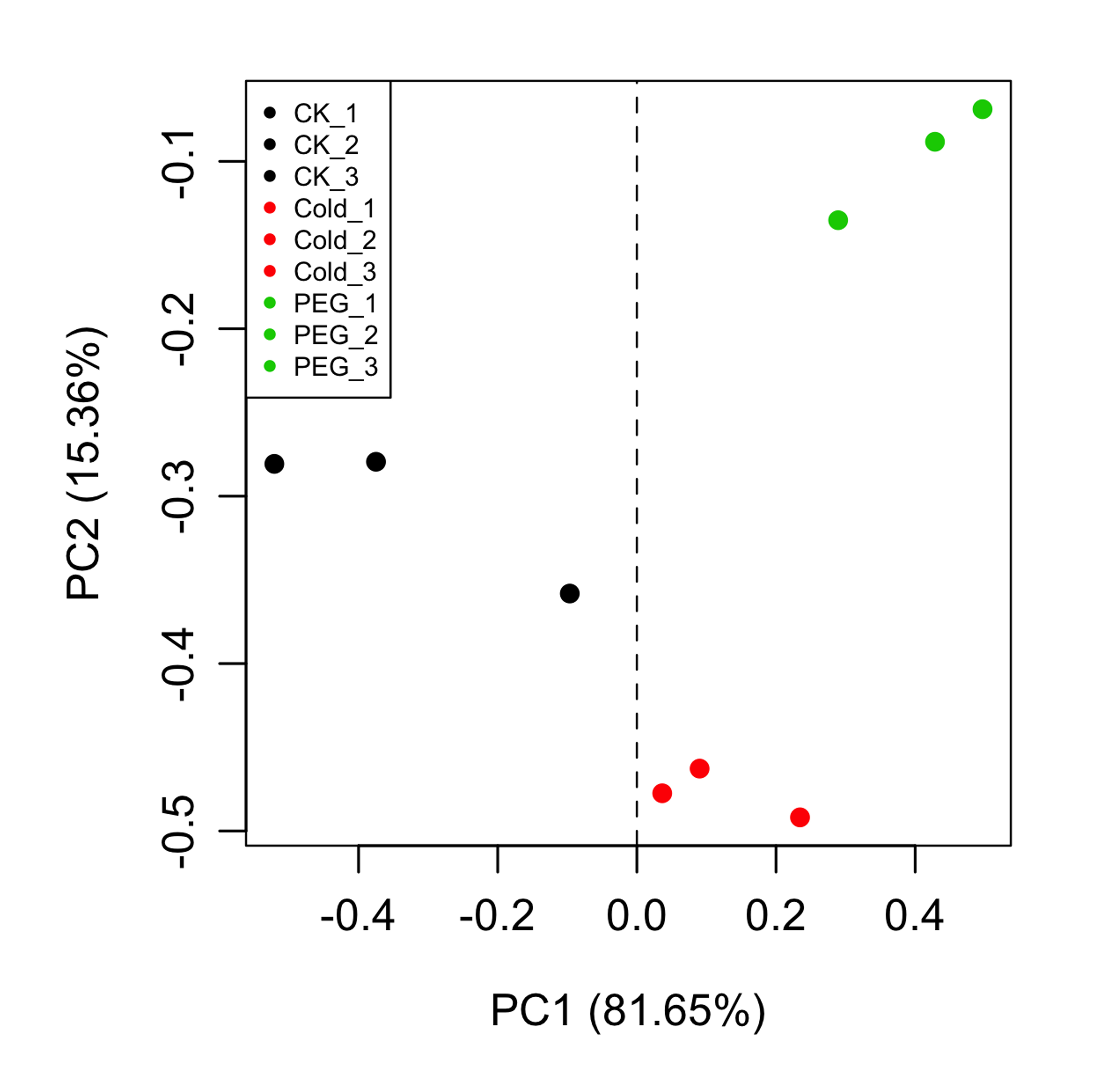

Supplement: FIGURE S1 — Principal component analysis of three samples for each treatment. [file Image_1.JPEG]
